# Research 101: A process for developing local guidelines for ethical research in heavily researched communities

**DOI:** 10.1186/s12954-019-0315-5

**Published:** 2019-07-01

**Authors:** Scott D. Neufeld, Jule Chapman, Nicolas Crier, Samona Marsh, Jim McLeod, Lindsay A. Deane

**Affiliations:** 1 Simon Fraser University, 8888 University Drive, Burnaby, V5A 1S6 BC Canada; 2 BC Centre on Substance Use, 400-1045 Howe St, Vancouver, V6Z 2A9 BC Canada; 3 Sex Workers United Against Violence, 334 Alexander St, Vancouver, BC V6A 1C3 Canada; 4 Megaphone Magazine Speaker’s Bureau, 312 Main St, Vancouver, BC V6A 2 T2 Canada; 5 Illicit: A Shadow Story, Vancouver, BC Canada; 6 Vancouver Area Network of Drug Users, 380 E Hastings St, Vancouver, BC V6A 1P4 Canada; 7 BC Yukon Association of Drug War Survivors, Vancouver, BC Canada; 8 Canadian Association of People Who Use Drugs, Vancouver, BC Canada; 9 Hives for Humanity, 206-312 Main St, Vancouver, BC V6A 2 T2 Canada

**Keywords:** Community-based research, Peer research, Local guidelines for ethical research, Empowering local knowledge, Community workshops

## Abstract

**Background:**

Marginalized communities often attract more than their share of research. Too often, this research benefits researchers disproportionately and leaves such communities feeling exploited, misrepresented, and exhausted. The Downtown Eastside (DTES) neighborhood of Vancouver, Canada, has been the site of multiple public health epidemics related to injection drug use as well as the site of much community-led resistance and struggle that has led to the development of cutting-edge harm reduction interventions (e.g., North America’s first supervised injection facility, Insite) and a strong sense of community organization. This background has made the DTES one of the most heavily researched communities in the world. Amidst ongoing experiences of unethical or disrespectful research engagement in the neighborhood, a collaboration between local academic researchers and community representatives developed to explore how we could work together to encourage more respectful, community-responsive research and discourage exploitative or disrespectful research.

**Methods:**

We developed a series of six weekly workshops called “Research 101.” These workshops brought together approximately 13 representatives from peer-based organizations in the DTES with a variety of experiences with research. Research 101 created space for community members themselves to discuss the pitfalls and potential of research in their neighborhood and to express community expectations for more ethical and respectful research.

**Results:**

We summarized workshop discussions in a co-authored “Manifesto for Ethical Research in the Downtown Eastside.” This document serves as a resource to empower community organizations to develop more equitable partnerships with researchers and help researchers ground their work in the principles of locally developed “community ethics.” Manifesto guidelines include increased researcher transparency, community-based ethical review of projects, empowering peer researchers in meaningful roles within a research project, and taking seriously the need for reciprocity in the research exchange.

**Conclusions:**

Research 101 was a process for eliciting and presenting a local vision of “community ethics” in a heavily researched neighborhood to guide researchers and empower community organizations. Our ongoing work involves building consensus for these guidelines within the community and communicating these expectations to researchers and ethics offices at local universities. We also describe how our Research 101 process could be replicated in other heavily researched communities.

**Electronic supplementary material:**

The online version of this article (10.1186/s12954-019-0315-5) contains supplementary material, which is available to authorized users.

## Background

Marginalized communities with high concentrations of poverty, substance use, housing issues, compromised health, and other expressions of the many brutal effects of historical trauma attract more than their share of research attention. Scholars^1^ have named these spaces “heavily-researched communities” [1], or “communities of over-studied Others” [2] and contend that the phenomenon of “over-research” [3] may often exacerbate and magnify the “territorial stigma” [4] associated with these places of “advanced marginality” [5]. The Downtown Eastside (DTES) neighborhood of Vancouver, Canada, is arguably such a place [6]. The DTES has been the site of multiple public health epidemics related to injection drug use (including multiple HIV/AIDS epidemics and overdose crises) and continues to be a space associated with illicit activities, impoverishment, and despair in the imaginations of researchers and the wider public alike [7–11]. To tell the story of the DTES in a different way, the neighborhood has also been the site of significant community-led resistance and struggle that has resulted in the development of cutting-edge harm reduction interventions to improve and save the lives of thousands (e.g., North America’s first supervised injection facility, Insite) as well as a strong tradition of community organization, peer-based engagement, and resident empowerment [12, 13]. The combination of these backgrounds has made the DTES one of the most heavily researched communities in the world [1]. There are many reasons why communities like the DTES attract so much research attention [3]. Researchers of all stripes are drawn to observe, document, and analyze the often public displays of oppression and suffering in the neighborhood. Activist academics and policymakers rally to evaluate and provide evidence for the effectiveness and economic efficiency of novel harm reduction efforts. Local universities liaise with the network of non-profits and peer-based organizations (i.e., organizations where the leadership and most of the membership are people with lived experience of poverty, homelessness, sex work, illicit drug use, etc.) operating in the DTES to funnel eager and compassionate undergraduate students into the neighborhood for research “experiences” in the eye-opening context of Vancouver’s poorest neighborhood. Too often, this research benefits researchers disproportionately and leaves communities like the DTES feeling exploited, misrepresented, and exhausted [1, 14].

A long tradition of academic critique, much of it initiated by indigenous scholars, has described and explained the historical tendency of research in marginalized communities (e.g., urban centers, indigenous communities, medicalized populations) to act as yet another exploitative arm of colonialism [15–17]. Research extracts data from marginalized communities in the form of bodily fluids, quantitative survey responses, and stories of personal pain/trauma, and rarely provides anything of much value in return (besides the odd ten-dollar gift card to the local grocery store). While much more could be said about the negative experiences of many DTES community members with research, this paper is not another account of the deficiencies of research in a heavily researched community. Rather, we describe the development of a partnership between university academics and DTES community members that formed to explore how we could work together to encourage more respectful, community-responsive research and discourage research that is exploitative, unhelpful, or disrespectful. We want to tell the story of our work together, the building of relationships, the planning and facilitation of a workshop series on ethical research in the DTES, and the resulting co-authored “Manifesto for Ethical Research” that we produced together because we believe a similar process could be replicated elsewhere. At its best, the process we describe has the potential to clarify a shared vision of “community ethics” based on the experiences and understandings of community members themselves. Better still, collaboration between community members and academic allies can translate these expectations for ethical treatment in the context of their neighborhood into guidelines for how researchers, and the research ethics boards (REBs) that evaluate their proposals, might begin to change their practices and effect systemic change within the landscape of community-based research.

## Methods

### The development of the “Research 101” workshop series

Research 101 began as an idea for a short “mini-course” on research methods to be offered free of charge to residents of the DTES. Research 101 was to be modeled on other “mini-courses” offered by non-profit organizations in the DTES neighborhood that present course material in a low-barrier setting, modifying traditional educational contexts to increase accessibility for low-income community members with varying degrees of comfort in formal educational settings and a wide range of literacy levels. SN’s hope was that by creating a course for DTES residents that would “introduce” them to a critical take on the research process this might get people talking about their own research questions about their community. Out of this context, a truly community-directed, participatory action research (PAR) project might emerge that SN could then help to facilitate as a part of his dissertation research. However, as SN began meeting with more people working in the DTES who had encountered issues of unethical or exploitative engagement from researchers, artists, and journalists alike, the idea for Research 101 evolved. Long-time community members and non-profit workers questioned how the initial Research 101 proposal assumed DTES community members were unfamiliar with critical issues surrounding research when they were in fact often highly experienced participants and co-investigators working alongside (or underneath) academic researchers. These early Research 101 collaborators also questioned the value of a lecture-style “course” rather than a more mutually constructed space for discussion and sharing between representatives from various research-involved organizations in the DTES. Research 101 was thus re-envisioned as a series of facilitated workshops for DTES organizations and community members to share their negative (and positive) experiences with research and express their hopes for how they would like to be treated by researchers instead. Similarly, SN’s role in the workshops evolved from that of a “course instructor” to a workshop facilitator and university insider, providing a framework for weekly discussions on different aspects of the research process as they pertained to community research in the DTES.

Importantly, Research 101 would not be a research project. Workshop contributors from the community would not be “research participants,” ethics approval would not be sought or obtained, and no “data” were to be collected or analyzed. Rather, we planned to undertake a collaborative effort together to discuss, summarize, and ultimately record a shared vision of “ethical research” from the perspective of DTES community members. We came to refer to this end product as an expression of “community ethics” which we defined as “a set of principles to guide behavior, based in lived experience, acknowledging the interconnectedness of our humanity, fostering relationships of respect, responsibility, reciprocity, and return.” [18]. To increase the usefulness of our shared vision of “community ethics” in research, we sought to develop shareable materials that could be adapted for a variety of DTES organizations, making it easier for them to evaluate research proposals more critically, set up processes that would protect the interests of their organization (e.g., developing template research agreements or research intake forms), and put the onus on researchers to do their own work in learning about issues with research in the DTES before they ever set foot in the DTES.

### Initial community engagement and workshop accessibility

Once a clear set of goals and vision for Research 101 was established, the work of finding workshop contributors and collaborators could begin in earnest. The goal of this community engagement process was to connect with as many peer-based organizations that engage with researchers in the DTES as possible. This process began as SN visited the weekly board meeting of the Vancouver Area Network of Drug Users (VANDU) to briefly describe the Research 101 workshop series and invite members of the board to participate. The problem of exploitative research in the neighborhood and the idea for the workshop series resonated deeply and several board members volunteered to participate. Importantly, at this meeting SN met SM, who proved to be both an eager Research 101 supporter (as well as an accomplished peer research assistant and published co-author on several academic papers) and a well-connected DTES community member with excellent networking skills. Through SM (and various other contacts in the DTES), SN was able to contact additional potential participants for Research 101 from the various boards and organizations SM was involved with, including a sex workers’ advocacy group and several other organizations comprised of people who use drugs in the DTES. These contacts either led to more board visits, where SN could briefly explain Research 101 and sign up additional participants, or to prominent individuals in the DTES community who expressed a direct interest in the Research 101 project and agreed to sign up on their own. This community engagement process of building relationships, showing up at community events and meetings, and personally inviting people to participate in Research 101 unfolded slowly over several months, resulting in a total of 18 potential participants (from 16 different DTES-related organizations) who had expressed an initial interest in attending Research 101. Of these, 13 participants came to at least one workshop and a smaller core group of 11 participants came to more than half of the workshops.

Workshops were designed to be as accessible as possible for participants with diverse backgrounds (including physical and mental health conditions, different relations to substances, low incomes/substandard housing, and various stigmatized identities) that sometimes made more traditional classroom or lecture-style learning environments uncomfortable. Community consultations early on made it clear that in order for the workshop to be accessible to many DTES community members, especially those with low incomes and regular use of illicit substances, there would need to be catered food and a weekly stipend (i.e., a cash payment of $20) to honor their time and contributions to the work of developing a shared vision of community ethics together. Workshops were held in a familiar meeting space in the DTES operated by a non-profit (“Hives for Humanity”) that was well-respected in the community. In order to set a sensitive and accessible tone for the workshop series, participants were encouraged to identify their accessibility needs (e.g., allergies, hearing/visual impairment, shyness, mobility need, etc.) in an intake survey at the first workshop. SN (the workshop facilitator) provided space for natural ground rules to emerge to guide subsequent discussions and help create a safe and comfortable environment for all. For example, participants were told to flag hurtful or oppressive language with a pre-determined hand signal and were explicitly encouraged to “call-out” the facilitator or fellow group members if discussions ventured into potentially triggering or retraumatizing territory. As much as possible, the workshop environment was responsive to individual needs and reactions as difficult subject matter was brought up or tensions among participants escalated, with SN facilitating through a trauma-informed lens that was sensitive to the complex relationships of each person in the room to exploitative research and intersecting marginalized identities. This responsive and flexible approach to facilitation meant that workshops also evolved over time. One memorable exchange took place in the aftermath of a disturbance created by an uninvited (and intoxicated) individual who joined the second workshop by simply walking in off the street. A workshop participant chided SN for allowing the individual in question to stay, thus putting others in the space at risk of harm or being “triggered”. She reminded SN that his outsider status in the DTES meant he did not know how meetings in the neighborhood are typically facilitated and recommended future workshops explicitly bar uninvited guests and include a speaker’s list and talking stick to reduce cross-talk and maintain meeting order. This participant’s recommendations were then implemented in the remaining Research 101 workshops.

### The shape of the Research 101 workshops

Six workshops took place from late February to April 2018. Each weekly 2.5-hour workshop (12:00–2:30) followed a similar pattern, with a low-key, catered lunch shared together before each workshop began. Lunch was followed by a welcome, indigenous territory acknowledgement, and time for each participant around the circle to check in and respond to an initial prompt related to the weekly topic (e.g., What is a positive or negative experience you have had with research?). Following this, SN would provide some brief context on the weekly topic (e.g., describing the usual process of a university ethical review and how some scholars have critiqued the suitability of university REBs for assessing the ethics of community-based research), taking care to translate academic jargon into language everyone could understand, and then pose several questions to stimulate discussion. Participants were encouraged to share stories of how the aspect of research under discussion that week could, in their experience, “go wrong.” After the first hour of workshop discussion, we would take a 10-min break and then spend the second hour of the workshop discussing expectations and hopes for more respectful or ethical researcher engagement on this aspect of the research process. Throughout the workshops, LD, an undergraduate research assistant would take notes to record participants’ insights and especially their ideas for better, more respectful research practice. These notes formed the basis of SN’s initial drafting of the “manifesto” summarizing the Research 101 discussions.

Content for each of the six workshops was informed by critical academic literature on ethics in community-based research and developed by SN in consultation with DTES community partners. Weekly themes were roughly based on the unique ethical issues that arise at different stages of a typical community-based research project. In the first week, extra time was spent acknowledging indigenous territories and situating the work of Research 101 within the wider context of research and colonialism. Extra time was also spent building connections between workshop participants, sketching out an overview of weekly topics to come, providing background information on the extent of research in the DTES and sharing personal stories of positive and negative relationships with research and researchers in the DTES. We also discussed possibilities for what we could create together as a way of summarizing the work of Research 101 and agreed at that point to create a “Manifesto for Ethical Research in the DTES.” Importantly, once Research 101 had been explained more fully for participants and the “Manifesto” had been agreed upon as a collectively produced end product, each participant was given a chance to verbally consent to their ongoing participation in the workshops. Everyone expressed their ongoing consent.

In the second workshop, we turned to the issue of first contact between researchers and the community, discussing how research first enters the DTES, the different types of researchers and research one might encounter (e.g., students, faculty, degree-based projects, grant-based projects, etc.), and how the institutional pressures of timelines, degree requirements, and promotions could sometimes entice otherwise well-intentioned researchers to violate their commitments to collaboration with community members in the interests of meeting deadlines. We discussed what kinds of details it might be important for a community organization to find out about a researcher before agreeing to work with them.

In the third week, we discussed the usual review processes of university REBs, the differences between universalized REB ethics codes and local conceptions of ethical research, and ways that community members could keep researchers accountable to their ethical commitments as described in their university ethics applications. We discussed the issue of consent in marginalized communities, engaged with different strategies for making consent an ongoing process rather than a one-time event, and brainstormed ideas for a community-based research ethics board in the DTES.

In week four, we discussed the various forms of community-based research that involve “peers” (i.e., people with lived experience of the research topic or community being researched) as co-researchers, and the ways that these important, and increasingly popular, approaches to field research can go wrong by replicating stigma and inequality within the research team. We considered different approaches to community-based research on a continuum of power sharing from academic researchers setting the agenda to community members creating and conducting their own research projects without any academic support or oversight.

In the fifth week, we discussed the meaning of reciprocity in community-based research and how researchers must not only ensure that research results are returned to the community in a meaningful fashion but explore further what it might look like for communities and researchers to benefit from research equally.

At the sixth and final workshop, SN brought hard copies of a rough draft of the “Manifesto” (based on LD’s weekly notes from workshops one to five) which were distributed to each participant and discussed as a group, line by line to arrive at a collaboratively produced edited draft of the Manifesto. In our concluding conversation on this final day, we also discussed our unifying goal of sharing the work of Research 101 and the Manifesto as widely as possible. We agreed to give each other permission to share the Manifesto in whatever spheres we could (e.g., academic conferences, DTES newsletters, magazine articles, word-of-mouth). We also agreed at the final workshop that the Manifesto was co-created by all workshop participants and that the names of everyone who participated in at least one workshop and who consented to being identified (some chose not to) would be listed alphabetically by last name as co-authors.

## Results

### Summary of the Manifesto

We have included a version of the full Manifesto text as a supplementary document (Additional file 1) to be read alongside this manuscript, but present a brief summary here. The Manifesto text is also available in its original (and regularly updated) format online at bit.ly/R101Manifesto [19].

The structure of the Manifesto follows the content of each week of Research 101 workshops closely. An initial section provides a brief description of the Research 101 workshops and the diverse organizational affiliations and backgrounds of workshop participants. Next, it describes the outcome of a brainstorming exercise conducted in the first week of workshops where examples of both the pitfalls and potential of research in the DTES were identified. This leads into not only an affirmation of the potential value of research in the DTES and the good intentions of most researchers, but also the need for researchers to recognize how the harms often associated with research are their responsibility to address. The Manifesto’s guidelines for what constitutes ethical research from the perspective of DTES community members are then positioned as a way for researchers to learn how to improve their ethical practice when working in the neighborhood.

A brief outline of the four primary sections of the Manifesto follows, closely mirroring weeks two to five of the Research 101 workshops and tracing the progression of a typical research project that might unfold in the community. The first section, “Getting to Know Each Other” deals with the issue of researcher transparency, especially in the early stages of setting up a potential research partnership, and provides a series of pointed questions to ask researchers about their identities and motivations for doing the research. The second section, “Ethical Review: Whose Ethics?” describes some of the limitations of university-based REBs in evaluating ethics applications for community-based research in the DTES. It also introduces the idea of a “Community Research Ethics Board” (CREB) based on the DTES that could enable community members themselves to review researchers’ ethics applications, an idea that is fleshed out in more detail with a series of recommendations for what this could look like in the context of the DTES. In the third section, “Doing the Research: Power and ‘Peers’” we summarize the many ways in which the positive development of researchers increasingly engaging “peer” researchers in their projects can go awry, and how this can be avoided. A final section on “Reciprocity and Bringing the Research Back” describes how infrequent and inadequate researchers’ attempts at knowledge translation or reciprocal exchange often are and how researchers could move beyond mere knowledge translation and begin to consider what true reciprocity might look like if community benefits from the research were equal to the benefits for researchers. The Manifesto concludes with a brief description of ways in which individuals and organizations can support the work of Research 101 and the Manifesto.

## Discussion

Several months after the Research 101 workshops had concluded and our draft Manifesto was looking increasingly polished (after having made the rounds between various community and academic colleagues for additional edits), co-author NC decided to write a personal letter in support of the Manifesto. He envisioned this letter as providing some of the wider context for the process of Research 101 and its significance for him and his wider community in the DTES. He hoped that it might help to emphasize the importance of the Manifesto and in this way help to convince local universities to take the Manifesto guidelines seriously and integrate them into REB policies for ethical review. In the letter, NC reflected on the significance of the great deal of research that takes place in the DTES:

I now know why we are so studied [in the DTES]. It is because our humanity, compassion and naturally cooperative impetus as a community is truly something to behold, even in its often uncomfortable images of impoverished or illicit instances, and if you ask me, it undeniably holds the key to our very survival as a species. For if we do not know how to be kind to one another, or how to communicate or share peacefully, when the world at large is intent upon isolating and incriminating us en masse, (apparently, for having the very brutal trauma and terror that they themselves have inflicted upon us) well then what will become of our sacred freedoms, our foundations as a society?

Indeed, the DTES is a target for so much research in part because of the incredible generosity of its people who, with great trust and vulnerability, have shared their often painful stories with researchers. Truly, these contributions from DTES community members over many years represent a gift to the world. The research knowledge amassed out of the DTES in the areas of harm reduction, HIV/AIDS, hepatitis C, injection drug use, heroin-assisted therapy, housing first policies, and overdose prevention (to name a few) is all thanks to the aggregated and anonymized offerings of generations of neighborhood residents who have given pieces of themselves to research [11].

Yet, this knowledge has come at a cost. NC’s letter eloquently expressed how data extraction feels like exploitation when DTES community members get the sense that they are merely facilitating the passage of knowledge from their lives and experiences to the upper echelons of academia and policymaking, producing knowledge which rarely circles back into the life of their community:

We peer community members and research participants in countless capacities, highly valued as wealths of firsthand knowledge of these ten square blocks known as the DTES, we're all pretty smart, but I don’t really think the other side, or rather the “research community”, actually cares as much about our involvement in research as we do, or at least I sense little in the way of real reciprocity. It’s really not fair that we consistently contribute our hearts and souls into this never-ending era of research run riot amongst our fragile neighborhood, currently mired in the grief of an ongoing overdose epidemic, yet still strong and responsive against unbelievable odds...but they’ll just smile and shake our hands, politely thank us for participating in whatever new thing their findings may very well conclude actually requires MORE research...and then they’ll go back to the other side and we'll probably never see them again, and they’ll not seek us out either. Not fair. Especially considering what I have witnessed them put people through in the name of research which, ultimately, might never have been possible WITHOUT our “lived experience” providing the guidance (of course, we never let the hurt show). Should we, the [peer] “experts”, as “hired help” not be at least recognized and fairly compensated? Perhaps it would be nice if we could even SEE the wider-scale changes and challenges come from all this hard won data-crunching and question-answering we are a part of inspiring, actually happen. See the very institution of academic, scientific, and/or media-related research itself openly acknowledge our humble little “hood”, our people, for being able to know exactly what the current issues are and also for being able (and willing) to articulate themselves in a tireless (and often quite eloquent) effort to give those issues a genuine voice.

As NC points out, researchers often raise the hopes of community members that by partnering their local expertise with academic expertise, they can produce knowledge to drive policy change that will better the lives of their fellow community members. In practice, however, community members are frequently disconnected from researchers once the project concludes, left to wonder what happened to the data, what conclusions might be drawn from it, and what policy changes advocated as a result.

This wider context of perceived injustice, disconnection and exploitation is why an intervention like Research 101 and the resulting Manifesto is necessary. NC’s letter went on to explain that it was only reasonable that the community respond by developing for itself, “a means of establishing a legitimate rights and responsibilities-based agenda for facilitating any and all forthcoming research endeavors in which our human neighborhood might ever become involved with and/or investigated upon. This is something research institutions require of themselves, that the researcher must first negotiate the scrutiny of an ethical committee of its senior echelon BEFORE a human subject is ever approached. Why should our own community not require the same level of scrutiny ourselves, being the ones who are studied?”

Empowering more, and more impactful, community scrutiny of research in the DTES is precisely what the Research 101 process was intended to achieve. While the grand universal ethical codes of Nuremberg, Geneva, Helsinki, and Belmont have been essential and instrumental in preventing innumerable research atrocities [20], they are no substitute for community oversight. Universal ethics codes have offered standardized, normative guidance for generations of researchers around the world attempting to toe the line between the human imperative to pursue knowledge, and the equally human imperative to care for others. However, these historical codes of universal (or “formal”) ethics, integrated into policies such as Canada’s Tri-Council Policy Statement on Ethical Conduct for Research Involving Humans [21] (TCPS2), are not sufficiently responsive to the unique histories and concerns of individual communities that have been harmed by research. Ethical codes have always been reactionary policy-making exercises. They are responses to some researcher atrocity of sufficient public significance (e.g., Nazi war experiments, the Tuskegee syphilis experiment, patient deaths in medical trials) that the research community was forced to bring greater clarity and standardization to the meaning of “ethical” research [17]. The value of Research 101 and the resulting Manifesto is precisely in its responsiveness to the immediate concerns of locally situated community members with deep experience of research and its effects (or lack of effects) in their home neighborhood. Such guidelines (or “practical ethics”) are invaluable, not only for empowering local residents and organizations to form more mutually beneficial partnerships with researchers, but also for university REBs and researchers who benefit from having a concise set of directions to guide them through the uncertain ethical terrain of community-based research in the DTES.

### Building support for the Manifesto

While we feel confident that the experiences and principles reflected in the Manifesto would resonate with a majority of DTES residents, we also know that the relatively small group of individuals who participated in Research 101 and helped to co-author the Manifesto do not speak for the neighborhood in its entirety [22–24]. Even though Research 101 participants came from a variety of backgrounds and different population-specific peer-based organizations in the DTES, these organizations did not specifically appoint participants as organizational representatives and the organizations themselves did not reflect the full range of diverse peoples and interests that comprise the DTES community. However, we have recently begun a strategic campaign of sharing the Manifesto widely within the DTES community in order to both inform residents of this new resource and to solicit official endorsements of the Manifesto from organizations and individuals. Putting the tools of the Manifesto into the hands of more neighborhood residents and organizations will hopefully increase their capacity to resist exploitative research and broker more equitable and mutually beneficial research partnerships. Furthermore, we believe that by continuing this process of community engagement and endorsement gathering, we can increase the validity of our claim that the guidelines laid out in the manifesto represent a widely shared vision of “community ethics” in the DTES.

We defined an “endorsement” to mean that an individual or organization supports the four key principles of the Manifesto: researcher transparency, community-based ethical review, peer empowerment in the research process, and reciprocity in the research exchange. We encouraged individuals and organizations to review the Manifesto carefully and make an informed decision as to whether they felt confident endorsing it. To date, the endorsement gathering process has involved tabling at local events related to the Manifesto (e.g., a recent official “launch” of a newly redesigned hard copy booklet version of the Manifesto, available online as well [25]) and reaching out to our existing networks of collaborators and contacts in the DTES to spread the word about the Manifesto and solicit endorsements. In some cases, organizations could review the Manifesto internally, consult with their staff team or board of directors, and respond with an affirmative endorsement quite quickly. Other organizations, especially peer-based organizations with boards comprised of neighborhood residents, have invited Manifesto co-authors to attend their meetings in order to provide a brief description of Research 101 (and the content of the Manifesto) and distribute copies of the Manifesto for members to review before they would support an endorsement. Several months into this process, we have managed to gather a total of 14 endorsements from significant organizations working closely with researchers in the DTES, including many of the organizations that had initially been engaged to recruit Research 101 participants. These include VANDU (the largest and most significant drug user organizations in the DTES), Sex Workers United Against Violence (SWUAV, a coalition of DTES-based current and former sex workers known for their outreach and advocacy), the Carnegie Community Centre (one of the largest community hubs in the DTES), and Pivot Legal Society (an activist law firm born out of a need to provide systemic level advocacy in support of the struggles of diverse peoples in the DTES). A full list of current endorsements is currently available in the online version of the Manifesto [19] and this will be updated regularly as more endorsements are added. Our community engagement and endorsement gathering process so far has been time-consuming, but we feel it is essential for both increasing community knowledge and utilization of the Manifesto and strengthening the validity of the Manifesto’s claim to represent a shared vision of community ethics in the DTES.

An additional five organizational endorsements (including the behavioral research ethics board of a local university) have come from university-based organizations that have research connections to the DTES, or from national or international organizations that collaborate with researchers (e.g., Toronto’s COUNTERfit Women’s harm reduction program or the Women’s Harm Reduction International Network). The Manifesto has also so far attracted over 54 individual endorsements from both individual residents of the DTES and faculty and students at local universities. This reflects our ongoing work of sharing about Research 101 and the Manifesto with fellow academic colleagues, researchers, and REB members at local universities. The onus of responsibility for changing the negative associations with research in the DTES rests most heavily on those creating the problems: researchers and the institutions that train them and approve their projects. Thus, we have also begun a process of knowledge mobilization and education on the Manifesto at local universities and research institutions in Vancouver to increase the likelihood that researchers planning new projects in the DTES will be guided by the principles laid out in the Manifesto. We also hope that DTES organizations themselves who are familiar with the Manifesto will increasingly distribute it to researchers who make requests to partner with them, directing them to follow its guidelines as a potential partnership unfolds. Indeed, we have already heard accounts of organizations using the Manifesto in this way.

### Integrating the Manifesto with local REBs

Ultimately, our goal is to integrate the principles of community ethics derived from the Research 101 process and laid out in the Manifesto with recognized REB review processes at local universities. This strategy will ensure that researchers are mandated to engage with the Manifesto guidelines and adapt their research plans accordingly to ensure community ethics in the DTES are being respected. In December 2018, this work took a significant step forward when co-authors SN, JC, NC, SM, and JM, along with community collaborators Sarah Common (Hives for Humanity) and Heather Holroyd (UBC Learning Exchange) met with representatives from three local REBs in Vancouver (University of British Columbia, Simon Fraser University, and Providence Health Care) to discuss the Manifesto and plans for integrating its principles into existing REB review policies. It was a lively meeting, with an overarching sense that solutions to address the problematic status quo of research in the DTES might be achieved through our ongoing collaboration as a diverse group of stakeholders. There were debates about terminology, questions about who did or did not have any money to support the ongoing work of outreach and support-building for the Manifesto and contrasting of the pros and cons of developing a Community Research Ethics Board (CREB) in the DTES independent from a university REB versus inviting DTES community members to join existing REBs when they were reviewing research related to the neighborhood. The meeting concluded with an acknowledgment of the need to build partnerships over time and that this would be the first of many meetings as we sought to work together to make research in the DTES more responsive to community concerns.

One possibility that continues to generate a great deal of enthusiasm among community members and academic allies alike is the formation of a Community Research Ethics Board (CREB) in the DTES. A CREB could act as an arbiter for “community ethics” in the DTES and an independent reviewing body that could supplement or work in tandem with existing university REBs when DTES-related proposals are submitted. We envision a process where a local university REB would flag any ethics proposals relevant to the DTES and forward these to the CREB, making the proposal’s continued movement through the university REB review process contingent on a successful CREB review. Trained DTES community reviewers could meet regularly to provide their feedback on ethics applications, ensuring community ethics are integrated into all future DTES-specific research. We are currently investigating different models for CREBs and exploring how a CREB might be developed, funded, and sustained over time. We have found inspiration in the pioneering work of Indigenous nations’ tribal REBs in the USA as well as the courageous example of the Bronx Community Research Review Board [26–28] and are exploring large-scale grant funding through a local foundation.

### The transferability of a Research 101 process to other heavily researched communities

One reason for describing the development of our locally situated, DTES-specific Manifesto for ethical research in an internationally distributed academic journal is because we believe that Research 101 is ultimately a process that could, and should, be replicated elsewhere. As the desire for research in communities that are already heavily researched is unlikely to abate, it may be best for these communities to take a “harm reduction” approach to the problem of over-research, asking themselves, “How could less research in our community be harmful and exploitative, and more research made to be collaborative, responsive and useful for us?” In some sense, ethics are always constructed culturally, and cultures differ as a function of local contexts and history. Above all, what may matter most of all is that a local process similar to Research 101 reflects the unique priorities and concerns of a given community. These idiosyncratic concerns with outside engagement emerge from the uniquely shared history of a place [29]. Rather than transplanting the principles from this Manifesto into other contexts, we suggest that the process itself is what could be transferred to another place most profitably by adapting this basic outline:

1. First, university allies or community members could start a process of community-engagement among individual community members or representatives of peer-based organizations in a given area to convene a conversation about recurring negative experiences with research and possible collective efforts to address these issues.
2. Second, we recommend creating a designated space (e.g., workshops, town hall meetings, research retreats, etc.) and time to bring together representatives from a wide variety of impacted organizations or sub-communities. This helps break down organizational silos and encourages local organizations to share with each other their best practices and insights (e.g., intake forms for outside requests, research agreement templates, good examples of positive collaborations with outsiders) for increasing community safety/empowerment and reducing community exploitation by outsiders.
3. Third, we recommend summarizing points of commonality in shared discussions on what constitutes an ethical engagement in some kind of document, report, or “manifesto.” A record of these discussions is useful for directing outsiders making future requests for engagement toward a widely shared vision of community ethics. A shared vision may also help discourage outsiders who do not get a favorable response from one organization from simply asking the next organization and the next until someone eventually caves to their requests for engagement.
4. Finally, once a common vision of “practical” research ethics has been developed, we recommend that it be shared widely, both within the community (to empower them in developing more equitable partnerships with outsiders) and with outsiders who engage with the heavily researched neighborhood (to educate them on community expectations for respectful treatment). Soliciting endorsements for the shared vision of community ethics may strengthen its claims to representativeness when endorsed by community groups and work as an accountability mechanism when endorsed by outside organizations.

## Conclusion

As NC observed in his Research 101 support letter, heavily researched communities are remarkable places; otherwise, they would not attract so much research attention. In the case of the Downtown Eastside of Vancouver, this neighborhood is remarkable for many reasons. Most commonly, it is represented as remarkable for its notoriety: the “poorest postal code in Canada” (it is not technically [30, 31]), the highest rate of HIV/AIDS in the developed world (at one time at least), Canada’s largest open drug scene, and so on. But these are representations of a place created and promoted almost exclusively by people who do not live there. Research is a powerful form of storytelling, and putting the research-setting agenda, and ethical compass to guide future research projects in the DTES firmly into the strong and capable hands of community members themselves may be an important way to begin to tell other stories of the Downtown Eastside more clearly.

## Additional file

Additional file 1: Research 101: A Manifesto for Ethical Research in the Downtown Eastside. (DOCX 51 kb)

## Data Availability

Not applicable. No “data” was collected as Research 101 was not a research study but a series of workshops. Research 101 “syllabus” description, logistics details, accessibility and expertise intake survey, and weekly outlines are available online at bit.ly/R101Materials

## Abbreviations

CREB: Community Research Ethics Board
DTES: Downtown Eastside
REB: Research Ethics Board (aka, an Institutional Review Board or IRB)
SWUAV: Sex Workers United Against Violence
TCPS2: Tri-Council Policy Statement: Ethical Conduct for Research Involving Humans
VANDU: Vancouver Area Network of Drug Users

## Footnotes

1 In general, our goal was to write this paper using colloquial language that is accessible to a diverse, non-academic audience. We therefore try to keep “academese” jargon to a minimum and include fewer academic citations than a typical academic paper might.
