# Supplementary material for: Research 101: A process for developing local guidelines for ethical research in heavily researched communities
Source: Harm Reduct J. 2019 Jul 1;16:41. doi: 10.1186/s12954-019-0315-5 (PMC6604375; doi:10.1186/s12954-019-0315-5)
Supplement: Supplementary file 1 — Research 101: A Manifesto for Ethical Research in the Downtown Eastside. (DOCX 51 kb) [file 12954_2019_315_MOESM1_ESM.docx]

**Research 101: A Manifesto for Ethical Research in the Downtown Eastside**

Co-Authored by:

Louise Boilevin, Jules Chapman, Lindsay Deane, Caroline Doerksen, Greg Fresz, DJ Joe, Nicolas Leech-Crier, Samona Marsh, Jim McLeod, Scott Neufeld, Steven Pham, Laura Shaver, Patrick Smith, Martin Steward, Dean Wilson, Phoenix Winter

Research 101 was a series of six weekly workshops (February to April 2018) to discuss research and ethics in the Downtown Eastside (DTES) neighborhood of Vancouver, BC, Canada. These workshops emerged out of a wider conversation on ethics in cultural production (e.g. research, media, artmaking) within the DTES convened by Hives for Humanity and supported financially with funding from Simon Fraser University’s Vancity Office of Community Engagement, also located in the DTES. Research 101 was but one of several components of this wider work to gather local knowledge and expertise on community ethics in the DTES into materials that could help empower the community^1^. Six to thirteen representatives from several diverse DTES organizations met each week to discuss their experiences with research, the wider context of research in the DTES, and community expectations for more ethical research practice.

The guidelines for ethical research presented in this “Manifesto” (available in its original form online^2^) are based on our discussions across six weeks of Research 101 workshops. They build on the work of the many other organizations, communities and individuals who have gone before us in expressing what it means for university researchers (and other people coming from outside the DTES community) to treat communities like the DTES with the respect and dignity they deserve, and expect. For example, the Vancouver Area Network of Drug Users (VANDU, the largest peer-based drug user activist organization in the world, operating in the DTES for over 20 years) has developed its own organizational policy^3^ to guide its partnerships with researchers entitled “Research and Drug User Liberation”. Similarly, the Vancouver Native Health Society (an Indigenous-focused health organization based in the DTES) evaluates research requests via their designated Research Committee which is guided by the principles of a Research Charter that was developed by the organization itself^4^. The Western Aboriginal Harm Reduction Society (WAHRS) has conducted its own community-based research on urban Aboriginal peoples’ experiences of research in the DTES^5^ and also developed a powerful research intake form that asks researchers a number of probing questions including why they want to work with the organization, what supports they will put in place to care for participants who may experience distress during the research, and how they plan to share their findings with the organization. This intake form has also been shared with and adapted for PACE Society, a peer-based DTES organization that employs and supports sex workers and sometimes engages with researchers as well. PACE has also collaborated with academic researchers to develop its own robust research ethics guide for community organizations^6^. In other examples, academic researchers have collaborated with peer-based organizations to reflect critically on what went wrong, and what could have gone better in specific research studies (e.g. Susan Boyd’s collaborative work with the “NAOMI Patient’s Association” or NPA^7^) or have conducted qualitative research on the perspectives of vulnerable populations on ethical research (e.g. Kirsten Bell and Amy Salmon’s research with women who use drugs^8^) and shared these in more formal, empirical journal article formats.

While no document or set of principles can truly represent the entire Downtown Eastside community in all its diversity^9-11^, Research 101 participants and Manifesto co-authors included peer leaders from mostly peer-based DTES organizations that represent and serve a variety of sub-populations in the DTES. These sub-populations include people who use illicit drugs and alcohol, Indigenous peoples, sex workers, people on methadone, low-income people and people living with mental health challenges (for a full list of workshop participants’ organizational affiliations see the online version of the Manifesto^2^).

**The Pitfalls and Potential of Research in the DTES**

There is no shortage of research in the DTES. Alina McKay, former graduate research assistant at the UBC Learning Exchange’s “Making Research Accessible Initiative”^12^ found that as of August 2017 there were 700 published research articles on the DTES, most since 2010 (personal communication). In 2016, there were 60 research articles published on the DTES and 67% of them were not made widely available to the public (i.e. you had to pay to download them). A systematic review of research literature published in 2012 found that 99 peer-reviewed articles, dissertations, and research reports on the DTES had come out between 2001 to 2011^13^.

Sometimes research in the DTES can be helpful, especially when done respectfully in true collaboration with the community. As co-author NC pointed out in a letter describing his experience of Research 101, the potential value of research is clear to many in the DTES:

*“Respect and reciprocity regarding research in the DTES is an idea that is growing and gaining both ground and momentum. This issue, speaking as a longtime resident of this community as well as an activist/advocate of its unified interests, is of the utmost importance, because research and all its often invasive and insensitive probing and placebo, is what gets results. And results matter. Research is what defines the bottom line or shows you there is no bottom. Research is what saves lives for some and for others makes life possible and for a fortunate few, research makes life very comfortable. Research manages the majority of our entire society and to just accept that so many [peer researchers] have sacrificed so much, for so long, for so little and have done so, so gracefully and without ever seeing so much as a thank you note, is not something, we as a legion of peer participants in these innumerable explorations, are willing or able to do.”*

Research can “get results” and contribute to positive social change. But research can also hurt. There are too many stories of organizations and individuals in the DTES who have felt disrespected by research^14^. Research can increase inequality, contribute to stigma^15,16^, exploit peoples’ pain^17^, exhaust community members^18-20^ and typically benefits researchers much more than it benefits the DTES^21-22^. In our first workshop together, we brainstormed some of the pitfalls and potential of research in the DTES, coming up with the list presented in Table 1.

*Table 1 – Research 101 Workshop #1 - Pitfalls and Potential of Research in the DTES*

| **Pitfalls** | **Potential** |
| --- | --- |
| Research can perpetuate stigma against people who use drugs, people who are low-income or homeless, sex workers etc. | Research can lead to positive change and actually benefit the community (e.g. research that helped keep Insite open) |
| Research can undermine community initiatives by ‘finding’ that they don’t fulfill certain, outsider-created expectations (when they meet important community needs) | Research can help educate and empower the community (especially if the questions being answered come from community members themselves) |
| Research can trigger trauma in participants with the kinds of questions researchers ask. | Research can help challenge stigma |
| Research can devastate people when researchers never return to share their results | Research can help complexify the issues (e.g. help people understand the wider story behind complicated social issues such as drug use) |
| Research can misrepresent communities and individuals, not give them an opportunity to respond or contest research findings | Research can help create evidence to support important community resources, harm reduction, Insite, heroin-assisted treatment (HAT) etc. |
| Research can be an excuse for not taking action on an issue, delaying action by ‘studying’ the issue instead | Research can create meaningful sources of income for people, help them learn new skills |
| Research can sap resources from communities, tie up highly capable community members in exhausting bureaucracy and prejudice so they have less to give in more meaningful community contexts |  |
| Research can be irrelevant to community members, and so detail-specific it feels unnecessary and not worth the valuable time and energy invested by community members |  |

We^a^ are not opposed to research. But we also reserve the right to refuse to participate in research^23^. We recognize that research has many pitfalls, but also feel hopeful about the great potential of research to create positive change. We recognize that researchers are not bad people. We believe that most researchers are trying to do the best work they can with the knowledge, experience and time that they have. However, we also believe that researchers can do better. In a time when the DTES continues to be hit hardest by the overdose crisis, housing crisis, and generations of colonial violence, we also continue to be inundated with research. The DTES should not have to bear the brunt of the costs associated with research. We should not have to continually exhaust ourselves working with researchers to ensure they are comfortable and acting respectfully within our community. We hope these guidelines can clearly communicate to researchers the expectations that DTES community members have for research that is respectful, useful, and ethical in the DTES community.

**What We Expect From Researchers Who Want to Work With Us**

In what follows, we break down the research process into four sections with different ethical issues we expect researchers to consider. First, when starting a research partnership, we want to know some things about who researchers are to ensure this work can start off in a good way. Second, during the initial phases of planning the research we want to subject research projects to our own community-based ethical review, in addition to the university-based ethics review process most research requires. Third, we expect researchers to include us in all aspects of the research process, and we have some expectations for how “peer” researchers can be included fairly and in ways that acknowledge the value of our unique expertise, including fair pay for our work. Finally, once the research is complete we expect that researchers will not just disappear, but will return to share their findings in a meaningful way with us and continue working together with us to turn research into action for positive change.

**1) Getting To Know Each Other**

Everyone has a history, the set of experiences that makes them who they are. Who researchers “are” as people is important knowledge for us to have when figuring out if we want to work with them or not. In the relationship between researchers and research ‘subjects’, we feel that the questions need to go both ways. If we can get to know you a bit beyond your identity as a researcher, that can also help make research feel less disconnected, and the whole experience feel more human. Oftentimes research can feel cold, impersonal, and dehumanizing – we want to try and make research a more human and equal exchange between people. If you want to do research with and about us, we want to know some things about you too:

1. Position: Who are you? Where are you from, what is your story and what is your connection to these unceded Indigenous territories? What are the types of social privilege (e.g. White? Male? Settler? Cis-gendered? etc.) you experience and how might those limit your ability to think and act ethically in your research?
2. Motivation: Why are you doing this research? Why do you have the research questions that you have? Could they be framed a different way? Are you asking the wrong questions? Who is your research for? Who doesn’t already know the answer to your research questions and why do they need to know? Is research “really the intervention needed”^24^ here and what makes you so sure?
3. Experience: What knowledge and experience do you have that makes you a good person to do this research with us in a sensitive and respectful way? If you have lived experience in the area of your research topic (e.g. substance use, sex work, homelessness, poverty) how has that affected your perspective on it?
4. Time: How much time do you have to complete this research? Is that enough time for you to collaborate with and include our community in the respectful way that we expect? Where are you at in the process of developing your research strategy? Is this project funded already? Have you received ethical approval from an REB already? When do you plan to start data collection? Is there still time/space for us to help shape the direction of this research?
5. Trauma-Informed: How do you plan to conduct your research in a way that is sensitive to peoples’ past experiences of trauma? How do you plan to create an environment of care and safety for community members working with you on your project, or participating in your research?
6. Politics: What are your politics, worldviews and opinions on the central issues of your research? What are the experiences you’ve had that have shaped these views? How might your politics affect your research, even in subtle ways?
7. Funding/Partnerships: What are your sources of funding and research partnerships? Who else is going to be seeing, or using, or benefitting from this research? What can you tell us about those people?
8. Feedback: What are your plans to return this research to the community in a respectful, meaningful and accessible way? How will you involve community members in data analysis and presentation of the results in a way that honours their unique expertise and your debt to them for making your research possible?
9. Reciprocity: How are *you* going to benefit from doing this research? How is the *community* going to benefit? Who is going to benefit *more*? How can we move towards reciprocity^22^ in the benefits for community and for you from this research? What are your plans for showing your thanks to the community in a meaningful way?
10. Action: What is your plan for taking action with us to advocate for change on the basis of your research? Is your research just another academic exercise creating more knowledge for privileged people to consume? If not, how are you going to include and empower us in your efforts to translate this research into action?

In addition to discussing these questions with you before working together on a research project, we might also find it valuable to hear from other people who have worked with you in the past. Is there someone (preferably a former research participant or peer researcher who worked with you) who could write you a “reference letter” to comment on your sense of research ethics, your respectfulness, and your personal integrity in conducting research in community settings?

**2) Ethical Review: Whose Ethics?**

Almost all university-based research with human subjects must receive ethical approval before researchers can begin data collection. While university REBs play an important role in making researchers consider and address the ethical issues raised by their research, they are not a guarantee of ethical research, at least by community standards. REBs are not equipped to keep researchers accountable to actually follow their proposed research protocols, REB members may not have much experience evaluating ethical issues in community-based (as opposed to laboratory) settings, and the universalized ethical codes REBs use to guide their evaluations (e.g. the “Tri-Council Policy Statement on Ethical Conduct for Research Involving Humans”^25^ or TCPS2 in Canada) typically prioritize ethical care for individual research participants over ethical care for communities or social groups^26^. REBs also might be using ideas of “risk” that make more sense to middle or upper class, White, settler folks than they do to community members^27-28^. Ultimately, we recognize that “ethics” are culturally and contextually constructed, and different people can have very different understandings of what “ethical” means. The ethical concerns of university REB members may not be the same as the ethical concerns of community members^27^.

At a bare minimum, we want to have a bit more information about the ethical review process you’ve gone through (or will go through) at your institution:

1. Can we read the ethics application you submitted to your university’s REB? Better yet, can you go through it with us and answer any questions we have about language, process, etc.?
2. How can we keep you accountable to following your ethics protocol? What options do we have for taking action if we notice some ethical issues with your research down the road? Who can we take a complaint to and what will the process be to resolve it?
3. Can we create a formal research agreement or “memorandum of understanding” that clearly lays out our expectations for you and your expectations for us as well as a clear plan for how we will work together throughout the process of your/our research in a way that meets our expectations for respectful and ethical engagement?

Here are a few areas where university REBs might not always consider the ethical concerns that are important in the DTES community^27^:

- Reciprocity^22^. It matters a lot to us that the community is benefitting from your research. Most of the time research benefits the academic researchers much more clearly and directly than it benefits community members. What is your plan to move towards this being a reciprocal relationship with mutual benefits for academic researchers and the community?
- Are you working from a trauma-informed perspective? How are you anticipating the harms to participants from discussing their pain or shame and what supports are you putting in place (e.g. counsellors) to care for people who might be triggered by participating in your research?
- Are your consent forms written in accessible language? How are you going to make absolutely sure that participants fully understand what you are asking them to do in your research?
- Even though we talk about “informed consent”, you can never fully predict what will happen in your research, and what the consequences will be, until it is underway or completed^29^. How are you going to make consent an ongoing^30^ process?
- Many research participants in the DTES have felt misrepresented (and exploited) by research^31^ (and media^32^, documentary film-makers^33-35^, artists^36-37^ etc.). Can you make a plan to let us review your interpretations and the context you’re putting around our words, stories and other “data” you’ve collected from our community? “member checking”^38^ will both help the validity of your research, and also give us more dignity and power in our relationship with you and your research.
- How are ethical considerations guiding everything about your research project? Are your research questions ethical? Is your research relevant enough to community interests to justify asking them to spend their limited time and energy helping you?
- What are the potential wider consequences of your research? What do you expect to find in your research? Who else could use the findings of your research and in what ways? Is it possible that other people will use your research to harm our community, or advocate for its elimination? How are you going to address this?

Even better than relying on university REBs, we want to work towards the creation of a Research Ethics Board based in the DTES community, modelled on the inspiring example of the Bronx Community Research Review Board in New York^39^. A Community REB (CREB) could be a powerful way of helping ensure more research meets our standards of community ethics in the DTES, and could serve a variety of useful functions for us in connecting academic researchers with the community in respectful and empowering ways.

In addition, we came up with a number of recommendations for how we envision the development of a CREB in the DTES:

- It should be housed in an accessible and familiar space that community members would feel comfortable accessing – a space of their own.
- It should have a clear mandate and process for involving community members as reviewers that is fair and empowers people with the tools and support they need to provide their expertise as critical thinkers with great concern for protecting the interests of their community.
- Training should be provided for community reviewers who have a lot of local expertise and care for the DTES community but may be less familiar with the process and ethical issues related to academic research.
- A CREB could have a rotating cast of reviewers, and/or a core group of more consistent reviewers with more familiarity with the process of ethical review of research.
- It could provide ways of enabling community members to see research proposals (e.g. post brief summaries in an accessible location) in advance of their review session and let people choose to review proposals that are especially relevant to their unique experience, interest and expertise.
- It should be based on the principles for ethical research in the DTES laid out in this Manifesto (and other community-created sources) and these could be posted in a visible location (e.g. a poster version of this Manifesto)
- It could increase researcher accountability and transparency by helping to keep track of research that has been reviewed in the community, the feedback that was given from the CREB, how researchers responded to that feedback, and the progress or outcomes of each research project that was reviewed.
- Parts of the CREB could be hosted online. Researchers could register for a review online and then be invited to an in-person review session after reviewers have had a chance to read and discuss their research proposal.
- As the CREB develops and members gains more experience, it could be integrated with the REBs at local academic institutions. Ideally getting your research reviewed by the DTES CREB (and responding to CREB feedback) would become a requirement for researchers who want to do their research in the DTES.

A CREB in the DTES could potentially serve as a hub for a few other helpful services:

- A space to coordinate education for researchers, and community members, on important ethical considerations when planning research in the DTES.
- A space that connects community members and organizations with research questions to university researchers with the resources to partner with the community to answer these questions through community-led, collaborative research.
- A CREB could provide a space, or paid position (e.g. a peer liaison or ombudsperson) to help mediate conflict between peer-researchers and academic-researchers^1^.
- A CREB could provide a space to disseminate and translate research on the DTES to community members (in conjunction with the Making Research Accessible Initiative at the UBC Learning Exchange^12^)

**3) Doing the Research: Power and “Peers”**

More and more, in response to movements like “Nothing About Us Without Us”^40-41^, researchers are involving community members in “peer researcher” roles within their community-based research projects. This is a valuable practice that has benefitted many community members. However, it can also raise new ethical issues as academic researchers can sometimes relate to their new colleagues from the community in ways that feel tokenizing, stigmatizing, or downright disrespectful^31^. Here are some expectations we have for “peer-based” research that could help researchers avoid disrespecting the peers on their research teams:

- Most basically, treat us with respect and dignity. Be polite. Recognize the value of our experience and expertise for your project. Show us gratitude. Praise us for our contributions, make us feel good about ourselves, just like you would for your colleagues in the university!
- Stop with the elitism. You might be an expert on some things but you’re not the expert on our lives or our community no matter how many years you’ve spent studying “addiction”, “homelessness”, “sex work” or anything else. “Don’t read us the book that we wrote”. Consider how the language you use, the way you dress, and other subtle cues from body language to tone communicate your sense of self-importance and how you see yourself in relation to us. Work with us in a spirit of humility instead.
- Stop with the entitlement. Research has been costly for our community, and very rarely has it actually benefited us. Don’t assume your research is helpful, wanted, needed, or going to be beneficial for our community. We don’t owe you anything.
- Be mindful of the inequality between academic researchers and community members^9^. Don’t act like it’s not there, but actively work to name it, and work towards creating conditions of actual equality between us in the research project. Think of a research team that includes academic and peer researcher in the terms of critical PAR researcher María Elena Torre^42^ as a “contact zone”, which she defines as “a messy social space where very differently situated people [can] work together across their own varying relationships to power and privilege” (p.110)
- Take seriously the actual meaning of the term “peer”. We’re not only peers/equals with our fellow community members. We are also peers with academic “experts” by way of the local expertise and experience that we bring to the project. “Peers” should mean that we are recognizing the unique expertise each person on the research team brings to the project, and trying to treat each other with equality^43^.
- Prioritize making this project accessible for community members. Provide the training we need to fully contribute our expertise to the project (e.g. designing research, collecting data, analyzing data, presenting findings). Work your schedule around community members, not academics.
- Work from a trauma-informed perspective^44^ that anticipates and avoids potential harms to us as a result of our collaboration with you. Recognize how common histories of trauma are in our community, and consider how working with you on your research project might impact us emotionally. Consider the risks we might be taking on in doing research in our community, with our friends and neighbors, and support us in self-care.
- Provide resources for peer researchers to support them in their lives beyond the research project. Become our friend and our ally.
- Make space for “peer” researchers to contest the way you’re developing the project, critique the way you’ve formulated the research questions, and speak back to your interpretations of the data. Recognize the value of our expertise and experience in this community in sharpening and improving your work.
- Give peers some actual power in the research project. “Tokenism” is a common experience where people feel like their peer position helps the research project look good on paper, but peers are actually given little power or space in the important decisions and work of the project. Nobody likes feeling tokenized. Don’t use us to check off a box that says you “included” the community^31^.
- Honour our ongoing work of survival and don’t shy away from necessary, though sometimes uncomfortable, conversations about money. Pay us fairly and promptly for our work on your project^45-46^. Don’t assume that we have nothing better to do or that it’s not a sacrifice to spend time working with you on a research project. Don’t assume we owe you something because of your ‘concern’ for our community. Don’t expect us to work for free. ALL of the time we spend with you working on your project needs to be compensated. Hustling for survival takes time, and if you take our time and don’t pay us we might need to hustle in ways that put us at more risk. Paying us cash is best too. We don’t ask what you do with your money, so don’t try and police what we do with our money either (i.e. please no more gift cards!).
- Try to recruit peers that are widely respected and trusted within their community. Recognize that certain people may not be the best representatives of the organization they are a part of^9^. Go through the proper channels for hiring people to work with you as representatives of their organization or community. Don’t just hire the first person you meet or have a connection with, or your friend in the community. They may not be the best person for the job, and not going through the proper channels to hire a peer research assistant could create bigger issues for your project down the road.
- Don’t perpetuate stigma in the way you work with us. Recognize the way that your own (even unconscious) prejudice towards people who use drugs, are homeless, engage in sex work, have poor health, or have low incomes might affect the way you work with us. It’s easy to notice, and really hurtful, when academics look down on us, don’t trust us, don’t want to empower us, act surprised when we say something intelligent or coherent and are generally condescending towards us. Use the opportunity of working with us to actively break down your own fears, prejudices and stigma. Get to know us as fellow human beings.
- If possible, connect us to a neutral third party mediator (a peer liaison or ombudsperson) who can help us navigate issues of power, disrespect, or inequality in the research relationship as things go along in order to help keep the project running smoothly and respectfully.

**4) Reciprocity and Bringing the Research Back**

Finally, once the research has been completed and the findings are ready to be shared, we expect that researchers will prioritize bringing their research back to the community^47^. In many people’s experience, this almost never happens, and if it does, it is entirely inadequate. It is not enough for you to email a copy of the academic article based on your research with us to the director of our organization. It is not enough for academic researchers to show up in our community with a PowerPoint presentation in language no one without a PhD can understand, expecting us to take on the work of booking a space, inviting people to come, providing food, and coordinating everything else that makes a community event accessible for folks in the DTES. Community feedback is your responsibility to initiate, organize and fund.

Here are a few basic considerations that are important for organizing a meaningful and accessible community feedback presentation:

- Organize all of the logistics for your presentation and do the work of inviting people, promoting the event, making it accessible (e.g. honoraria, translation, child care if necessary).
- Translate academic language and concepts into relevant and comprehensible descriptions for a wide audience (e.g. different levels of literacy, non-native English speakers)
- Provide a meaningful territorial acknowledgement where your work is contextualized in the context of unceded territories, resilient Indigenous communities and centuries of Indigenous resistance to colonization. Pay a representative from a local First Nation to provide a welcoming.
- Enable meaningful involvement of participants/community members in the event, or in the presentation of the research – what did this research mean to them?
- Organize community-friendly food that people can easily eat and digest. For example, some people in our community don’t have all of their teeth and can’t exactly eat a crunchy carrot salad...
- Book a community space that is comfortable and accessible for people (e.g. in the neighborhood, in a familiar building). Have someone there who can de-escalate conflict (i.e. a peer or community member) if necessary. Support people who might be triggered emotionally by hearing about your research.
- Show appropriate gratitude to your participants, your collaborators, and the community at large for contributing to your research.
- Make it an opportunity to receive feedback on your developing interpretations of the data. Give participants a sense of the way you plan on contextualizing their words and stories and encourage people to challenge your interpretations or speak back to your interpretations. This is not only respectful, it makes for better research^38^.
- Use the presentation as a jumping off point for future possible research collaborations, more involvement from community members in your ongoing work, and a foundation for more community-led research and action.
- Make it clear how your research can lead to action that will help the neighborhood and how you will support community members in their struggles for positive change.
- Provide cash honoraria for people helping at the event, perhaps even for attendees
- Make your presentation a celebration of community strength, resilience, and dignity
- Make it clear that you want to maintain ongoing relationships with the people that helped you do the research. Invest in long term relationships, don’t just break ties as soon as the research is completed.

In addition to a community presentation of your research here are some other ways of providing meaningful knowledge translation in the community:

- Co-write a community research report with your community collaborators that prioritizes only topics of interest and relevance for the community and is written entirely in accessible language. Do this before you move on to working on more technical academic reports based on the research.
- Write plain language summaries of your published research (though maybe check with some community members first to ensure they are truly written in “plain” language...) and make these available online or in hard copies at the relevant organizations or community spaces where you did your research.
- Invite participants or co-researchers to present with you on your research at conferences, in academic guest lectures to your colleagues or in university classes etc. Provide the support and resources for community members to fully participate with you in sharing about the research.
- Find ways of inviting co-researchers to present your work in impactful formats to policy makers and politicians. Don’t be afraid of turning your research into action research. Often community members know the best people or organizations to direct your research and advocacy towards.

While bringing the research back is a basic expectation most community members have, it is also not enough. While people may be interested in the research findings, they are often more interested in reciprocity^22^. At this stage in the research when the benefits for academic researchers are becoming clear (e.g. publications, grant money, degrees, jobs), the benefits for the community need to be prioritized as well.

A few suggestions for ways of building reciprocity in the research relationship:

- Provide a cash donation to the organization you worked with as a way of saying thank you and recognizing the value of their work.
- Volunteer your time and skills to contribute to the work of the organization. Don’t be afraid of contributing in simple ways such as volunteering in the kitchen. Use these opportunities to build relationships and gain an understanding of the wider community.
- Organize a community event that benefits children in the neighborhood, or lifts up peoples’ voices and stories.

**Supporting the Manifesto**

We are currently looking for personal and organizational endorsements of the Research 101 Manifesto. You can indicate your support for the Manifesto by adding your name to our google form here: bit.ly/R101Support. An endorsement means that you/your organization agrees with the Manifesto principles of Researcher Transparency, Community-Based Ethical Review, Peer Empowerment in the Research Process and Reciprocity in the Research Exchange. Read through this manifesto closely, consult with your organization (especially organizations that participate in cultural production in the DTES or DTES organizations that work with researchers, journalists etc.), and sign the google form or contact us (Scott Neufeld, sdn2 [at] sfu.ca) if you might be able to arrange an organizational endorsement of the Manifesto. We would then add your name/organization to a list hosted on the online version^2^ of the Manifesto at bit.ly/R101Manifesto.

**Endnote**

^a^We chose to write the Manifesto in the “voice” of DTES community members who participated in Research 101 (i.e. not the university-based workshop participants and facilitators) and whose ideas and suggestions for how they wanted researchers to treat them provided the bulk of the content for the Manifesto. Thus, the words “we” and “us” refer to the DTES community members who participated in Research 101. The manifesto is addressed towards “you”, the academic researcher.

**References**

1 Hives for Humanity. Empowering Informed Consent – Community Ethics in Cultural Production. <http://hivesforhumanity.com/communityethics/> Accessed 31 January 2019.

2 Boilevin L, Chapman J, Deane LA, Doerksen C, Fresz G, Joe D, Leech-Crier N, M

arsh S, McLeod J, Neufeld SD, Pham S, Shaver L, Smith P, Steward M, Wilson D, and Winter P. Research 101: A Manifesto for Ethical Research in the Downtown Eastside. Updated as of April 11, 2019. <http://bit.ly/R101Manifesto>. Accessed online 12 April 2019.

3 Vancouver Area Network of Drug Users. Research and Drug User Liberation. <http://www.vandu.org/research/> Accessed online 12 April 2019.

4 Vancouver Native Health Society. Vancouver Native Health Society Research Committee Charter Prepared By VNHS Research Committee. <http://www.vnhs.net/downloads/charter-finalized-2.pdf> Last updated 3 February 2015. Accessed 31 January 2019.

5 Western Aboriginal Harm Reduction Society. Talking circle series: Urban Aboriginal peoples’ experiences with research. <http://wahrs.ca/wp-content/uploads/2017/03/Aboriginal-Peoples-Experiences-with_Research.pdf> Accessed 31 January 2019.

6 Bowen R for PACE Society. Research ethics: A guide for community organizations (working draft). <http://www.pace-society.org/wp-content/uploads/2014/04/Community_Research_Guidelines.pdf> Last revised February 2006. Accessed 31 January 2019

7 Boyd S, NAOMI Patients Association. Yet they failed to do so: Recommendations based on the experiences of NAOMI research survivors and a call for action. Harm reduction journal. 2013 Dec;10(1):6.

8 Bell K, Salmon A. What women who use drugs have to say about ethical research: findings of an exploratory qualitative study. Journal of Empirical Research on Human Research Ethics. 2011 Dec;6(4):84-98.

9 Boyd SC. Community-based research in the Downtown Eastside of Vancouver. Resources for feminist research. 2008;33(1/2):19.

10 Jewkes R, Murcott A. Community representatives: representing the “community”?. Social Science & Medicine. 1998 Apr 1;46(7):843-58.

11 Walden B. Community research mythology. The Qualitative Report. 2006;11(1):55-79.

12 The University of British Columbia. Making Research Accessible Initiative. <https://learningexchange.ubc.ca/community-based-research/making-research-accessible-initiative/> Accessed 31 January 2019.

13 Linden IA, Mar MY, Werker GR, Jang K, Krausz M. Research on a vulnerable neighborhood—the Vancouver Downtown Eastside from 2001 to 2011. Journal of Urban Health. 2013 Jun 1;90(3):559-73.

14 McMartin P. The high cost of misery in Vancouver’s Downtown Eastside. Vancouver Sun. January 5, 2016. http://www.vancouversun.com/health/pete+mcmartin+high+cost+misery+vancouver+downtown+eastside/11632586/story.html

15 Liu S, Blomley N. Making news and making space: Framing Vancouver's Downtown Eastside. The Canadian Geographer/Le Géographe Canadien. 2013 Jun;57(2):119-32.

16 Woolford A. Tainted Space: Representations of Injection Drug-Users and HIV/AIDS in Vancouver's Downtown Eastside. BC Studies: The British Columbian Quarterly. 2001(129):27-50.

17 Tuck E. Suspending damage: A letter to communities. Harvard Educational Review. 2009 Sep 1;79(3):409-28.

18 Way E. Understanding research fatigue in the context of community-university relations. Local Knowledge: Worcester Area Community-Based Research. Paper 3. http://commons.clarku.edu/localknowledge/3

19 Clark T. We're Over-Researched Here!' Exploring Accounts of Research Fatigue within Qualitative Research Engagements. Sociology. 2008 Oct;42(5):953-70.

20 Culhane D. Domesticated time and restricted space: University and community women in Downtown Eastside Vancouver. BC Studies: The British Columbian Quarterly. 2003(140):91-106.

21 Bell K, Salmon A. What women who use drugs have to say about ethical research: findings of an exploratory qualitative study. Journal of Empirical Research on Human Research Ethics. 2011 Dec;6(4):84-98.

22 Maiter S, Simich L, Jacobson N, Wise J. Reciprocity: An ethic for community-based participatory action research. Action research. 2008 Sep;6(3):305-25.

23 Tuck E, Yang KW. R-words: Refusing research. Humanizing research: Decolonizing qualitative inquiry with youth and communities. 2014:223-48.

24 Tuck E. Biting the University That Feeds Us. dissident knowledge in higher education.:149-167.

25 Canadian Institutes of Health Research, Natural Sciences and Engineering Research Council of Canada, and Social Sciences and Humanities Research Council of Canada. Tri­Council Policy Statement: Ethical Conduct for Research Involving Humans. December 2014. http://www.pre.ethics.gc.ca/pdf/eng/tcps2-2014/TCPS_2_FINAL_Web.pdf Accessed 31 January 2019.

26 Flicker S, Travers R, Guta A, McDonald S, Meagher A. Ethical dilemmas in community-based participatory research: Recommendations for institutional review boards. Journal of Urban Health. 2007 Jul 1;84(4):478-93.

27 Tuck E, Guishard M. Uncollapsing ethics: Racialized sciencism, settler coloniality, and an ethical framework of decolonial participatory action research. Challenging status quo retrenchment: New directions in critical qualitative research. 2013:3-27.

28 Bell K, Salmon A. Good intentions and dangerous assumptions: Research ethics committees and illicit drug use research. Research Ethics. 2012 Dec;8(4):191-9.

29 Bell K. Resisting commensurability: Against informed consent as an anthropological virtue. American Anthropologist. 2014 Sep 1;116(3):511-22.

30 Culhane D. Stories and plays: Ethnography, performance and ethical engagements. Anthropologica. 2011 Jan 1:257-74.

31 Damon W, Callon C, Wiebe L, Small W, Kerr T, McNeil R. Community-based participatory research in a heavily researched inner city neighbourhood: Perspectives of people who use drugs on their experiences as peer researchers. Social Science & Medicine. 2017 Mar 1;176:85-92.

32 Mackie J, Wood S.E. Can reporting on vulnerable people do more harm than good? The Tyee. July 12, 2018. https://thetyee.ca/Mediacheck/2018/07/12/Reporting-Do-Harm-Good/

33 Youdan, M.P. Kickstarting the Gentrification Movement: How Gnarcore leveraged the DTES in their search for funds. The Mainlander. April 16, 2014. http://themainlander.com/2014/04/16/kickstarting-the-gentrification-movement-how-gnarcore-leveraged-the-dtes-in-their-search-for-funds/

34 Walls R. *Visibility in Vancouver: screen stories and surveillance of the Downtown Eastside* (Doctoral dissertation, University of Nottingham).

35 Sepulveda Martinez JM. Procedural residues after the shooting of The Ballad of Oppenheimer Park. Unpublished Masters of Fine Arts Thesis. http://summit.sfu.ca/item/14173

36 Parkatti, K. When an outsider paints the DTES missing women. Vancouver Observer. February 9, 2011. https://www.vancouverobserver.com/politics/investigations/2011/02/08/when-outsider-paints-dtes-missing-women

37 Butler ML. The Hero of “Heroines". Mosaic: A Journal for the Interdisciplinary Study of Literature. 2004 Dec 1:275-96.

38 Simpson A, Quigley CF. Member checking process with adolescent students: Not just reading a transcript. The Qualitative Report. 2016;21(2):376-92.

39 Bronx Community Research Review Board. [www.bxcrrb.org](http://www.bxcrrb.org) Accessed 31 January 2019

40 Jürgens R. " Nothing about us without us"-greater, meaningful involvement of people who use illegal drugs: a public health, ethical, and human rights imperative. Canadian HIV/AIDS Legal Network; 2005.

41 Canadian HIV/AIDS Legal Network. Nothing about us without us: A manifesto by people who use illegal drugs. https://www.opensocietyfoundations.org/sites/default/files/Intl%2520Manifesto%2520Nothing%2520About%2520Us%2520%2528May%25202008%2529_0.pdf Accessed 31 January 2019.

42 Torre ME. Participatory action research and critical race theory: Fueling spaces for nos-otras to research. The Urban Review. 2009 Mar 1;41(1):106-20.

43 Canadian AIDS Society. Peerology: A guide by and for people who use drugs on how to get involved. <http://librarypdf.catie.ca/PDF/ATI-20000s/26521E.pdf> Accessed 31 January 2019

44 US Department of Health & Human Services. Substances Abuse and Mental Health Services Administration – Trauma informed approach and trauma-specific interventions. <https://www.samhsa.gov/nctic/trauma-interventions> Accessed 31 January 2019.

45 BC Centre for Disease Control. Peer payment standards for short-term engagements: Created in collaboration with peers and providers. <http://www.bccdc.ca/resource-gallery/Documents/Educational%20Materials/Epid/Other/peer_payment-guide_2018.pdf> Last updated February 2018 V1. Accessed 31 January 2019.

46 Greer AM, Pauly B, Scott A, Martin R, Burmeister C, Buxton J. Paying people who use illicit substances or ‘peers’ participating in community-based work: a narrative review of the literature. Drugs: Education, Prevention and Policy. 2018 Sep 3:1-3.

47 Ontario Centre of Excellence for Child and Youth Mental Health. Knowledge mobilization plan. http://www.kmbtoolkit.ca/sites/all/themes/kmb/assets/images/Knowledge_Mobilization_Planning_Form.pdf Accessed 31 January 2019.
